# Intermittent fasting-associated microbiome remodeling and functional modulation by Lacticaseibacillus rhamnosus HN001

**DOI:** 10.3389/fnut.2026.1947132

**Published:** 2026-09-24

**Authors:** Tinghui Shao, Jin-Ming Liu

**Affiliations:** 1 Department of Pathology, Nanjing Drum Tower Hospital, Affiliated Hospital of Medical School, Nanjing University, Nanjing, China; 2 CERVO Brain Research Centre, Québec City, Québec, Canada; Department of Psychiatry and Neurosciences, Faculty of Medicine, Université Laval, Québec, QC, Canada

**Keywords:** gut microbiome (GM), intermittent fasting (IF), Lacticaseibacillus, Lacticaseibacillus rhamnosus HN001, metabolomics

## Abstract

**Objective:**

The gut microbiome is a critical regulator of host metabolism, yet how intermittent fasting (IF) remodels the gut ecosystem independent of dietary composition remains unclear. This study aimed to isolate the effect of feeding rhythm on the gut microbiome and identify specific microbial taxa mediating IF-induced metabolic changes.

**Methods:**

We employed a family-based ‘shared meal’ model that strictly standardized dietary composition across all participants. Five healthy adults (3 males, 2 females) from a single family participated in a pilot, hypothesis-generating 4-week 16:8 IF intervention with longitudinal fecal sampling for 16S rRNA sequencing and untargeted metabolomics. To functionally test a candidate Lacticaseibacillus-associated mechanism, C57BL/6 J mice were subjected to a 1-week 16:8 IF protocol with or without daily oral supplementation of Lacticaseibacillus rhamnosus HN001. Plasma beta-hydroxybutyrate (betaOHB) and peptide YY (PYY) were measured by ELISA.

**Results:**

IF was associated with body weight loss in humans (2.1 +/− 0.4 kg, 95% CI: 1.3 to 2.9 kg, *p* < 0.05) without a significant reduction in caloric intake. In the primary paired genus-level DESeq2 analysis accounting for participant identity, no genus remained significant after false-discovery-rate correction, although Lacticaseibacillus showed a directional decrease after IF, consistent in direction with the original exploratory unpaired analysis. Predicted microbial functional and fecal metabolomic changes were consistent with altered microbial energy metabolism. In mice, IF significantly elevated plasma betaOHB (*p* = 0.0046) and reduced body weight, whereas HN001 supplementation reversed these IF-associated effects under the conditions tested.

**Conclusion:**

In this pilot, hypothesis-generating human cohort, IF was associated with coordinated microbiome and metabolomic remodeling, including a directional Lacticaseibacillus signal that motivated subsequent functional testing. In mice, *L. rhamnosus* HN001 supplementation reversed IF-associated weight loss and ketosis, supporting the functional plausibility of a Lacticaseibacillus-associated mechanism. The human observations require confirmation in larger, independent cohorts with time-matched non-fasting controls and species-resolved microbiome measurements.

## 1 Introduction

Intermittent fasting (IF) has gained prominence for its efficacy in weight management and metabolic improvement (1, 2). A hallmark of IF-induced weight loss is its biphasic trajectory: an initial period of rapid reduction is invariably followed by a substantial slowdown, often culminating in a sustained plateau (3–5). This adaptive attenuation points to the engagement of systemic regulatory mechanisms that extend beyond a simple caloric deficit. While the host’s metabolic shift toward lipid oxidation and adjustments in appetite-regulating hormones such as peptide YY (PYY) are recognized contributors (6–10), the precise biological triggers that orchestrate the distinct temporal dynamics of weight loss—particularly the decisive early phase—remain poorly understood.

The gut microbiota, a dynamic microbial interface exquisitely sensitive to dietary rhythms (11, 12), is a prime candidate for mediating the metabolic effects of IF (13, 14). It critically influences host energy harvest, substrate metabolism, and inter-organ signaling (15–17). However, in human studies, a fundamental confounder has obscured clear mechanistic insight: the dominant effect of dietary composition on microbial community structure often overwhelms the subtler signal of changes in feeding timing (18, 19). Consequently, specific microbial taxa and pathways that are directly reprogrammed by the fasting rhythm itself, rather than by concurrent dietary changes, have remained elusive.

To examine the effect of intermittent fasting on the gut ecosystem under standardized dietary conditions, we conducted a controlled pilot study with longitudinal multi-omics profiling. The human data were treated as hypothesis-generating. Genus-level analysis identified a directional Lacticaseibacillus signal alongside broader predicted functional and metabolomic changes consistent with altered microbial energy metabolism. Because V3-V4 16S sequencing cannot definitively resolve closely related Lacticaseibacillus species, the human data do not establish species-specific depletion of *L. rhamnosus*.

Despite substantial evidence linking IF to metabolic benefits, three fundamental questions remain unresolved. First, the relative contribution of the gut microbiome versus caloric restriction to IF-induced weight loss is unknown, as most human studies have not rigorously controlled dietary composition, making it impossible to isolate the effect of feeding rhythm from dietary content. Second, it remains unclear whether IF-induced microbial changes are merely correlates of metabolic improvement or functional drivers of the phenotype. Third, the specific microbial taxa and molecular pathways that mediate IF’s metabolic effects have not been identified, limiting the potential for microbiota-targeted interventions. To address these gaps, we adopted a family-based ‘shared meal’ model that strictly standardizes dietary composition across all participants, thereby reducing dietary-composition variability and facilitating assessment of longitudinal changes associated with feeding rhythm. We then integrated longitudinal multi-omics profiling to identify IF-responsive microbial and metabolic signatures. Finally, we translated the human findings into a targeted causal intervention in mice—supplementing a representative Lacticaseibacillus strain, *L. rhamnosus* HN001 during IF—to determine whether this single taxon is functionally sufficient to reverse IF’s metabolic effects (i.e., to test functional rescue rather than establish necessity). This ‘human discovery-to-animal validation’ paradigm was designed to test for a causal contribution in a direction that cannot be tested in human studies alone.

These observational findings led us to test the functional plausibility of a Lacticaseibacillus-associated mechanism in mice. *L. rhamnosus* HN001 was used as a representative, experimentally tractable Lacticaseibacillus strain to test functional sufficiency—whether supplementation could modify the metabolic response to IF—rather than as a confirmed replacement for a species definitively identified as depleted in the human cohort. This design therefore separates the exploratory human association from the controlled mouse intervention and does not establish that depletion of endogenous *L. rhamnosus* is necessary for the human phenotype.

## 2 Methods

### 2.1 Mice

Mice were group housed (2–3 mice per cage) in standard polycarbonate cages under a 12:12 h light–dark cycle (lights on at 07:00) with ad libitum access to water and standard chow unless noted otherwise. Room temperature was maintained at 22 ± 2 °C and relative humidity at 50 ± 10% throughout the experimental period. All animals were acclimatized to the housing conditions for at least 1 week prior to the start of the experiment. Mice were kept in an EVC cage with nesting material. Mice were routinely monitored for undesirable infection agents. The C57BL/6 J mice were obtained from Experimental Animal Center of Nanjing Drum Tower Hospital. All of the experiments were conducted in accordance with the Regulations for the Administration of Affairs Concerning Experimental Animals (China) and were approved by the Nanjing Drum Tower Hospital Animal Ethics Committee (Approval No.: 2025–0676-01).

The mice were subjected to a 16:8 intermittent fasting protocol. This regimen consisted of a daily 16-h fasting period followed by an 8-h ad libitum feeding window. The protocol was maintained for a duration of 1 week. To translate the human 16:8 IF protocol (4 weeks) to mice, we accounted for interspecies differences in metabolic rate and circadian biology. Mice have an approximately 7-fold higher mass-specific metabolic rate than humans and a correspondingly faster gut transit time (20, 21). Additionally, mice are nocturnal feeders; thus, the 16-h fasting period was aligned with their active (dark) phase to maintain circadian alignment with the human protocol where fasting occurs during the awake period. This translational framework has been previously validated (22). Specifically, the 16-h fasting period for mice was scheduled from 19:00 to 11:00 (dark-to-light transition), and the 8-h feeding window from 11:00 to 19:00 (light-to-dark transition), ensuring that fasting coincides with the naturally active phase in both species. For mice, food was provided ad libitum during the 8-h feeding window for IF groups and throughout the 24-h period for the Ctrl group. Caloric intake was measured daily at the cage level to assess whether differences in feeding quantity contributed to the observed weight changes. Pre-weighed chow was provided at the beginning of the feeding period, and remaining food (including spillage) was weighed at the end of the feeding period. Food intake was measured daily at the cage level (n = 3 cages per group, 2 mice per cage). Daily measurements from each cage were averaged over the 7-day intervention period to generate a single mean value per cage (g food per cage per day), which served as the independent observation for statistical analysis. No normalization to body weight was applied.

### 2.2 Human subjects

This study enrolled five healthy adults (3 males, 2 females) with a mean age ranging from 30 to 35 years. All participants had no history of gastrointestinal or metabolic diseases and had not taken antibiotics, prebiotics, or probiotic supplements in the 3 months prior to the study initiation. The study protocol was reviewed and approved by the Institutional Ethics Committee of the participating hospital (Approval No.: 2023–648-03). Written informed consent was obtained from all participants. This study did not perform sex- or gender-based analyses, as the sample size (*n* = 5, with 3 males and 2 females) was insufficient for any meaningful subgroup comparison. The inclusion of both sexes was intentional to reflect the natural demographic composition of adult family units and to enhance the generalizability of findings beyond a single-sex cohort. However, we acknowledge that sex-dependent differences in gut microbiota composition and metabolic responses to fasting may exist and cannot be assessed in this pilot cohort. All participants were analyzed as a single group, and the reported findings represent sex-averaged responses. The research adhered to the ethical principles for medical research involving human subjects as outlined in the Declaration of Helsinki.

A within-subject pre-post design was adopted, wherein each participant served as their own control. This design was chosen based on the well-established principle that inter-individual variation in gut microbiota composition substantially exceeds intra-individual variation over time (23). By comparing each participant’s post-IF microbiome and metabolome to their own baseline, we effectively control for the dominant sources of inter-individual variability—including genetic background, habitual diet, and environmental exposures—that would otherwise confound a parallel-group design. This approach maximizes sensitivity to detect IF-induced changes within the same individual, which is particularly important in a small-sample exploratory study focused on deep multi-omics phenotyping.

Five healthy subjects from the same family voluntarily undertook a 4-week intervention consisting solely of 16:8 intermittent fasting (IF). Body weight was measured before and after the IF intervention, and BMI was calculated. The IF protocol restricted daily energy intake to an 8-h window (e.g., 7:00 a.m. to 3:00 p.m.), permitting only non-caloric water consumption during the remaining 16 h. Throughout the study, participants consumed the same types of foods without prescribed caloric restriction, allowing ad libitum intake within the 8-h feeding window. This design was chosen to mimic real-world IF conditions, where individuals may spontaneously reduce caloric intake due to the restricted feeding window. To quantify this, daily food records were maintained throughout the study. To further eliminate inter-individual dietary variation as a confounding factor, a standardized meal provision model was implemented for the entire study duration. All daily meals for participants voluntarily undertaking the IF regimen, including three main courses and any permitted snacks, were uniformly prepared and delivered by a central kitchen designated by the research team. The daily menu was designed by a certified nutritionist to ensure nutritional balance. Although minor adjustments were made based on family preferences (e.g., avoidance of specific allergens), the macronutrient composition and total caloric intake remained strictly consistent. Participants were instructed to consume only the study-provided food and water and to refrain from ingesting any other food or caloric beverages.

During the four-week intervention period, participants were contacted weekly via telephone or a secure messaging application to monitor adherence to the fasting protocol and standardized diet, assess any adverse events, and provide general support. Compliance with consuming the study-provided meals was verified through daily self-reported checklists and random audits of meal delivery records. No participants voluntarily undertaking the IF regimen withdrew from the study.

#### 2.2.1 Sample size consideration

Given the exploratory and proof-of-concept nature of this highly controlled feeding study, which required enrolling entire families and providing standardized meals—a logistically intensive and costly design—a formal *a priori* sample size calculation was not performed. The sample size of five healthy participants from a single family was chosen to maximize homogeneity (controlling for genetic and environmental confounders) and to demonstrate the feasibility of the ‘family meal’ model for isolating the effects of feeding rhythm. Notably, the effect sizes for IF-induced weight loss (Cohen’s d = 0.25 for body weight, d = 0.33 for BMI) were calculated and Cohen’s d was reported as a descriptive standardized effect-size measure. We report these effect sizes as descriptive measures of the magnitude of change, but we emphasize that a small effect size does not itself demonstrate adequate statistical power or exclude false-positive findings. Given the very small sample size, these results should be interpreted with caution and require replication in larger cohorts. The primary aim was to generate robust mechanistic hypotheses through deep multi-omics phenotyping in a tightly controlled setting, which can be tested in larger, statistically powered trials in the future. The results of this proof-of-concept study should be interpreted as hypothesis-generating rather than definitive, and external validation in independent, larger cohorts is required. We emphasize that the absence of a time-matched non-fasting control group means that temporal variation, behavioral changes, adaptation to the standardized diet, environmental factors, or regression to the mean cannot be excluded as alternative explanations for some of the observed changes.

#### 2.2.2 Study setting, timeline, and procedures

This study was conducted at a single tertiary care center. All standardized meals were prepared and delivered from a designated central kitchen within the study institution. All anthropometric measurements (body weight, body mass index) and fecal sample collections were synchronized with the respective intervention phases, performed at baseline (Day 0) and upon completion (Day 28) of each intervention period.

#### 2.2.3 Anthropometric assessment (body weight and BMI)

Body weight was measured to the nearest 0.1 kg using a calibrated electronic scale. Participants were instructed to wear light indoor clothing and no shoes during measurements. Height was measured to the nearest 0.1 cm using a wall-mounted stadiometer. BMI was calculated as weight in kilograms divided by height in meters squared (kg/m^2^). All anthropometric measurements for all participant groups were performed by the same trained research staff at the participating hospital, following an identical standardized protocol at baseline (Day 0) and endpoint (Day 28) of each intervention period.

#### 2.2.4 Assessment of adherence

Adherence to the 16:8 intermittent fasting (IF) protocol was monitored using daily self-reported logs, in which participants recorded their fasting and feeding start and end times. Compliance with the consumption of the standardized study-provided meals was verified through daily checklists submitted by participants and reviewed during weekly check-in sessions with a study dietitian. Participants were instructed to report any deviations from the prescribed fasting schedule or consumption of non-study foods or caloric beverages immediately.

#### 2.2.5 Statement on comparability of assessment methods across groups

To ensure the validity of comparisons between study groups, all outcome assessments—including anthropometric measurements (body weight, height, BMI), fecal sample collection, handling, storage, and subsequent laboratory analyses (16S rRNA gene sequencing, non-targeted metabolomics)—were performed using strictly identical protocols, equipment, reagents, and personnel.

### 2.3 Fecal sample collection

Fecal samples were collected from each participant at two time points: before the initiation of the IF intervention (Day 0) and upon its completion (Day 28). Fresh stool specimens were self-collected by participants using sterile containers, promptly transported to the laboratory under cold chain conditions, and subsequently stored at −80 °C until further analysis.

### 2.4 Non-targeted metabolomics analysis

#### 2.4.1 Metabolite extraction

Exactly 100 mg of each fecal sample was homogenized with pre-cooled methanol/water extraction solution (v/v, 4:1) using a tissue homogenizer. Following centrifugation, the supernatant was collected, subjected to vacuum freeze-drying, and then reconstituted in 80% methanol for subsequent LC–MS analysis.

#### 2.4.2 Chromatography-mass spectrometry analysis

Metabolite separation was performed using an ultra-performance liquid chromatography (UPLC) system (Thermo Scientific) equipped with an ACQUITY UPLC HSS T3 column (2.1 mm × 100 mm, 1.8 μm). Mass spectrometric detection was carried out on a Q Exactive HF-X mass spectrometer (Thermo Scientific) operating in both positive and negative ionization modes.

#### 2.4.3 Data processing and identification

Raw data were processed using Progenesis QI software (Waters, USA) for peak alignment, peak picking, and normalization. Metabolites were preliminarily identified by matching their accurate mass-to-charge ratios against the Human Metabolome Database (HMDB) and the Kyoto Encyclopedia of Genes and Genomes (KEGG). Multivariate statistical analyses, including principal component analysis (PCA) and orthogonal partial least squares-discriminant analysis (OPLS-DA), were performed using the MetaboAnalystR package. Differential metabolites were screened based on the following criteria: variable importance in projection (VIP) > 1 from the OPLS-DA model and *p*-value < 0.05 from Student’s t-test. No additional multiple-testing correction was applied to the metabolomics data, as this was an exploratory analysis; findings should be interpreted as hypothesis-generating. Pathway enrichment analysis of differential metabolites was conducted using the KEGG and HMDB databases. For quality control, pooled QC samples were injected every 10 runs to monitor instrument stability. Missing values were imputed using half of the minimum detected value. Data were normalized by total signal intensity and log-transformed prior to analysis. OPLS-DA model performance was evaluated using R^2^ and Q^2^ values; model robustness was assessed by 200-fold permutation testing. The potential for model overfitting was considered given the small sample size (n = 5 per group), and results were interpreted conservatively. Metabolite identifications were based on accurate mass and retention time matching against HMDB and KEGG databases (Level 2 identification confidence).

### 2.5 16S rRNA gene sequencing and bioinformatic analysis

#### 2.5.1 DNA extraction and amplification

Total genomic DNA was extracted from approximately 200 mg of each fecal sample using the QIAamp Fast DNA Stool Mini Kit (Qiagen, Germany). The V3-V4 hypervariable region of the bacterial 16S rRNA gene was amplified via polymerase chain reaction (PCR) with universal primers 341F (5′-CCTAYGGGRBGCASCAG-3′) and 806R (5′-GGACTACNNGGGTATCTAAT-3′).

#### 2.5.2 Library construction and sequencing

The resulting PCR amplicons were purified, quantified, and subjected to paired-end sequencing (PE250) on an Illumina MiSeq platform (Illumina, USA).

#### 2.5.3 Data processing and analysis

Raw sequencing data were processed using the QIIME2 pipeline (version 2020.11). Briefly, the DADA2 plugin was employed for quality filtering, denoising, and chimera removal to generate a table of amplicon sequence variants (ASVs). Taxonomic assignment of representative ASV sequences was performed against the SILVA database (version 138) using the classify-sklearn naïve Bayes classifier with default parameters. The V3-V4 region used in this study supports genus-level interpretation, whereas definitive species-level assignment among closely related Lacticaseibacillus taxa is limited; accordingly, species-level inferences were treated cautiously. Based on the final ASV table, alpha diversity (Chao1 and Shannon indices) and beta diversity (Bray-Curtis distances visualized by PCoA) were assessed. The primary genus-level differential-abundance analysis was performed on raw counts using DESeq2 with a paired design that explicitly accounted for repeated measurements from the same participant (design = ~ subject + condition). Participant identity was included as a blocking factor and condition represented pre- versus post-IF sampling; the primary contrast was post-IF versus pre-IF. *p* values were adjusted using the Benjamini-Hochberg false discovery rate (FDR), with adjusted *p* < 0.05 considered statistically significant. For transparency, the originally performed unpaired DESeq2 analysis (design = ~ condition) is retained as an exploratory sensitivity analysis but is not used for primary statistical inference. Functional predictions were generated from the ASV table using PICRUSt2 (v2.5.0) against the KEGG Orthology database (release 2023.01), normalized by copy number, and analyzed using STAMP (v2.1.3). Differential abundance of predicted KEGG pathways between pre- and post-IF groups was assessed using Welch’s t-test with Benjamini-Hochberg FDR correction (q < 0.05). These are predicted functions based on 16S-derived taxonomic profiles, not direct measurements of microbial gene expression or enzymatic activity.

### 2.6 Lacticaseibacillus rhamnosus HN001 supplementation in mice

The probiotic strain used in this study was HOWARU® Lacticaseibacillus rhamnosus HN001™ (IFF Health Sciences). HN001 was administered by oral gavage at 6 × 10^8^ CFU per mouse per day. This dose was selected within the range commonly used in murine probiotic intervention studies (10^8^–10^9^ CFU per mouse per day) (24, 25). Fresh probiotic preparations were suspended in sterile PBS daily immediately before administration. Viability was confirmed by plate counting on MRS agar prior to the experiment (>90% viable bacteria at administration). No dose–response titration was performed.

### 2.7 Measurement of plasma β-hydroxybutyrate (βOHB) and peptide YY (PYY) by enzyme-linked immunosorbent assay (ELISA)

#### 2.7.1 Sample collection and preparation

Blood samples were collected from mice via the retro-orbital plexus at the endpoint of the experimental interventions. Samples were immediately transferred into pre-chilled heparin sodium tubes and gently inverted several times to prevent coagulation. Plasma was separated by centrifugation at 2,000 × g for 15 min at 4 °C, aliquoted into sterile microcentrifuge tubes, and stored at −80 °C until analysis. Prior to ELISA, plasma samples were thawed on ice and centrifuged at 10,000 × g for 10 min at 4 °C to remove potential precipitates.

#### 2.7.2 ELISA procedure

Commercially available sandwich ELISA kits were used for the quantitative determination of β-hydroxybutyrate (βOHB; Mouse β-hydroxybutyrate ELISA Kit, Catalog No. YFXEM00642, YIFEIXUE BIO TECHNOLOGY) and peptide YY (PYY; Mouse Peptide YY ELISA Kit, Catalog No. YMS2646-B, YIFEIXUE BIO TECHNOLOGY). All assays were performed according to the manufacturers’ protocols. Plasma samples were diluted 1:5 with the provided sample diluent prior to analysis. Absorbance was measured at 450 nm using a microplate reader, and sample concentrations were interpolated from standard curves generated with serial dilutions of the provided standards using a four-parameter logistic (4-PL) curve fit. All samples from the same experimental cohort were analyzed on the same plate to minimize inter-plate variability; the coefficient of variation (CV) for duplicate samples was maintained below 15% (9, 26).

### 2.8 Definition of variables

The primary exposure was the 16:8 intermittent fasting (IF) regimen, defined as a daily 8-h feeding window (e.g., 07:00–15:00) followed by a 16-h fast where only non-caloric water was permitted. The secondary exposure was the concurrent administration of the probiotic *Lacticaseibacillus rhamnosus* HN001.

The primary outcomes were changes in absolute body weight (kg) and body mass index (BMI, kg/m^2^), measured at baseline (Day 0) and endpoint (Day 28). Exploratory mechanistic outcomes included shifts in gut microbiota composition (via 16S rRNA gene sequencing) and the fecal metabolomic profile.

Key potential confounders were identified and addressed. Dietary composition, a major potential confounder in nutrition studies, was standardized through the meal-provision model, thereby reducing dietary-composition variability across the intervention. The use of antibiotics, prebiotics, or probiotics within 3 months prior to the study and a history of gastrointestinal or metabolic diseases were exclusion criteria. Other potential confounders such as physical activity levels, sleep patterns, and stress were acknowledged but not formally measured or controlled, as the primary aim was to investigate longitudinal changes associated with IF under standardized dietary conditions.

### 2.9 Bias mitigation

Several strategies were employed to minimize potential biases. Selection bias was mitigated by enrolling members of the same household, ensuring highly homogeneous backgrounds. To address measurement bias, all laboratory personnel conducting 16S rRNA sequencing and metabolomic analyses were blinded to participant group and time point. Confounding bias from dietary composition was rigorously controlled through the standardized meal provision. Detection bias for the primary outcome (weight) was mitigated by using calibrated equipment and a standardized measurement protocol, although the assessors were not formally blinded to the intervention phase. Adherence was monitored via self-report, which may introduce reporting bias; future studies would benefit from objective monitoring tools.

### 2.10 Quantification and statistical analysis

All experiments and data analyses were conducted blindly. Statistical comparisons were performed using SPSS 20.0 software with appropriate inferential methods, with t data presented as the mean ± s.e.m. For independent two-group comparisons, normally distributed data were analyzed using two-sided unpaired t-tests. For longitudinal human pre-post comparisons, within-subject dependence was accounted for using paired tests. The non-normally distributed data were analyzed by Mann–Whitney U-tests for two-group comparisons and by the Kruskal-Wallis test and Dunn’s multiple comparisons test for more than two groups. Statistical significance was set at *p < 0.05, **p < 0.01, ***p < 0.001, and ****p < 0.0001. ns, no significance. Cohen’s d was reported as a descriptive standardized effect-size measure.

All continuous variables were assessed for normality using the Shapiro–Wilk test. Variables that did not meet the normality assumption were log-transformed prior to parametric analysis; if transformation did not achieve normality, non-parametric tests were employed as stated. No missing data were present for the primary outcomes. Potential outliers were identified using the ROUT method (Q = 1%) and were retained in the primary analysis; sensitivity analyses excluding outliers were conducted and confirmed the robustness of the findings.

For longitudinal human pre-post comparisons, within-subject dependence was accounted for using paired tests where applicable. For the primary 16S genus-level differential-abundance analysis, participant identity was explicitly included as a blocking factor in DESeq2 (design = ~ subject + condition). The original unpaired DESeq2 analysis was retained only as an exploratory sensitivity analysis (Supplementary Table S2) and was not used for primary statistical inference. Complete results from the primary paired analysis are provided in Supplementary Table S1. Given the pilot cohort size (*n* = 5 paired participants), human taxon-level findings that did not survive FDR correction were interpreted as exploratory and hypothesis-generating.

For the mouse experiments, group comparisons were performed using pre-specified comparisons with unpaired two-tailed t-tests for the following pairs: Ctrl vs. IF (to assess the effect of IF), IF vs. IF+HN001 (to assess the effect of HN001 during IF), and Ctrl vs. Ctrl+HN001 (to assess the effect of HN001 under normal feeding conditions). No additional multiple-testing correction was applied given the limited number of pre-specified comparisons and the distinct hypotheses tested for each pair. For food intake data measured at the cage level (n = 3 cages per group), the cage mean was used as the independent observation for statistical comparisons using unpaired t-tests for the same three pre-specified comparisons.

### 2.11 Analysis of subgroups and interactions

Given the limited sample size of this pilot study, pre-specified formal subgroup analyses were not feasible and were therefore not performed.

### 2.12 Sensitivity analyses

To assess the robustness of our primary findings, we performed the following sensitivity analyses:

Outlier handling: We re-analyzed the body weight change data using non-parametric tests (Wilcoxon signed-rank test) and after excluding potential outliers identified by the ROUT method (Q = 1%). The significance of the IF-induced weight loss remained unchanged.

Adherence-adjusted analysis: For the IF intervention, we performed a per-protocol analysis including only participants with high adherence (≥90% compliance based on self-reported logs). The effect size and direction of weight change were consistent with the primary intention-to-treat analysis.

Microbiome analysis robustness: the primary genus-level inference was based on the paired DESeq2 model accounting for participant identity. The original unpaired DESeq2 analysis and alternative exploratory analyses were retained as sensitivity analyses; concordant directionality was considered descriptive and was not used to override the FDR-corrected paired result.

Sensitivity analyses were interpreted as exploratory and did not replace the paired primary analysis.

## 3 Results

### 3.1 Intermittent fasting significantly reduces body weight in human subjects

Previous studies have indicated that intermittent fasting can improve metabolic health and lead to weight loss (2, 3, 27). Given that weight loss during IF is most pronounced in the first 1–2 months and subsequently slows significantly (3–5). To investigate whether the gut microbiota participates in the host’s metabolic adaptation during the initial rapid weight loss phase of intermittent fasting (IF), we selected members of the same family as study subjects. This approach ensured identical dietary composition and meal timing, thereby eliminating dietary variation as a confounding factor for gut microbiota changes (28–31). The study participants self-implemented a 4-week 16:8 intermittent fasting intervention (Figure 1A). Subject characteristics are shown in Figure 1B. The results demonstrated that IF led to a progressive reduction in body weight (Figure 1C). After 4 weeks, both body weight and BMI were significantly lower compared to measurements taken before the IF intervention (Figures 1D,E). After the 4-week IF intervention, body weight decreased by an average of 2.1 ± 0.4 kg (mean ± SEM; 95% CI: 1.3 to 2.9 kg; range: −1.5 to −2.8 kg), from a baseline of 68.2 ± 5.1 kg to 66.1 ± 4.9 kg (*p* < 0.05). Accordingly, BMI decreased by 0.7 ± 0.1 kg/m^2^ (95% CI: 0.5 to 0.9 kg/m^2^), from 23.1 ± 1.2 to 22.4 ± 1.1 kg/m^2^ (*p* < 0.05). Despite the constrained sample size—an inherent feature of our tightly controlled, homogenized dietary regimen—the effect sizes for body weight (Figure 1D; d = 0.25) and BMI (Figure 1E; d = 0.33) were calculated. We report these effect sizes as descriptive measures of the observed changes. However, we emphasize that statistical inference from this small cohort is limited and requires replication in larger, independent cohorts. To determine whether IF-induced weight loss was associated with reduced caloric intake, we analyzed daily food records maintained throughout the intervention. Total daily energy intake during the IF period was comparable to baseline (Figure 1F). Notably, this observation was made during the early phase of IF (4 weeks in humans), coinciding with the period of most rapid weight loss; whether a similar dissociation persists during chronic IF remains to be determined. However, these human findings are derived from a small exploratory cohort without a non-fasting control group, and should be interpreted as hypothesis-generating.

**Figure 1:**
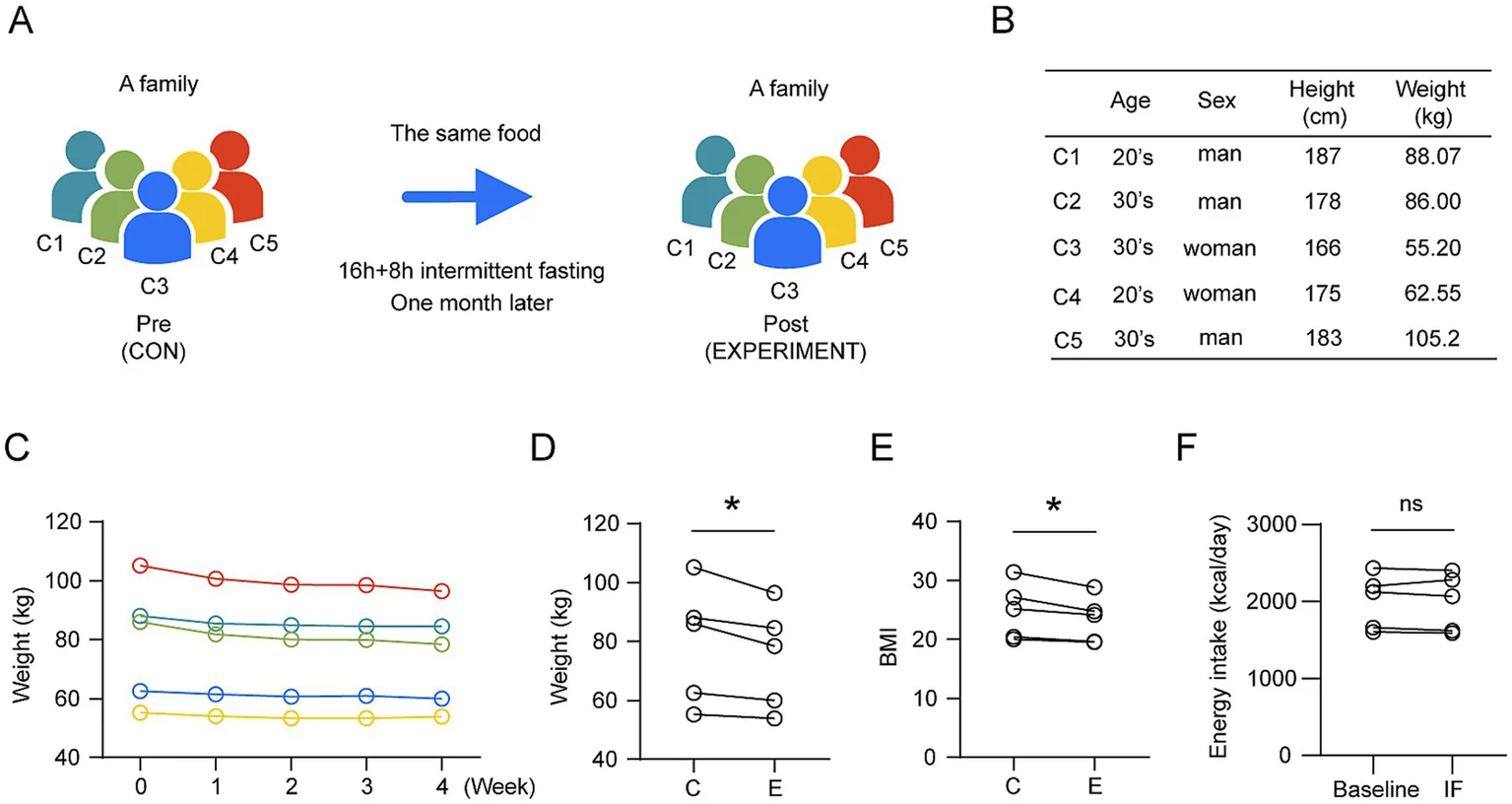
Intermittent fasting significantly reduces body weight in human subjects. **(A)** Schematic diagram of 16:8 intermittent fasting under identical dietary conditions. **(B)** Subject information sheet. **(C)** Body weight changes in subjects following 4-week intermittent fasting. **(D)** Body weight before (C group) and after (E group) 4 weeks of intermittent fasting (C: *n* = 5, E: *n* = 5, *p < 0.05). **(E)** BMI before (C group) and after (E group) 4 weeks of intermittent fasting (C: *n* = 5, E: *n* = 5, *p < 0.05). **(F)** Daily en ergy intake at baseline and during the 4-week IF intervention. Each line connects paired measurements from the same individual. *n* = 5. Data are shown as individual values with mean ± SEM. ns, not significant (paired t-test: *p* = 0.5517, t = 0.6491).

### 3.2 Intermittent fasting alters fecal metabolomic profiles consistent with altered gut luminal and microbial metabolism

To investigate metabolic changes in human subjects before and after intermittent fasting under controlled dietary conditions, we collected fecal samples from participants both pre- and post-IF intervention for untargeted metabolomic analysis (Figure 2A). Principal component analysis revealed distinct alterations in metabolite composition between the pre- and post-intervention groups (Figures 2B,C). We further identified differentially abundant metabolites between the two groups (Figures 2D,E). As summarized in Table 1, the upregulation of Octadecanamide and Docosatrienoic acid suggests enhanced lipid metabolism, while the downregulation of Heptethylene glycol implies a reduction in glucose metabolism (Table 1). These fecal metabolomic changes are consistent with a pattern of reduced carbohydrate-related metabolites and increased lipid-related metabolites. However, as these data reflect gut luminal metabolism rather than systemic substrate flux, this interpretation remains indirect.

**Figure 2:**
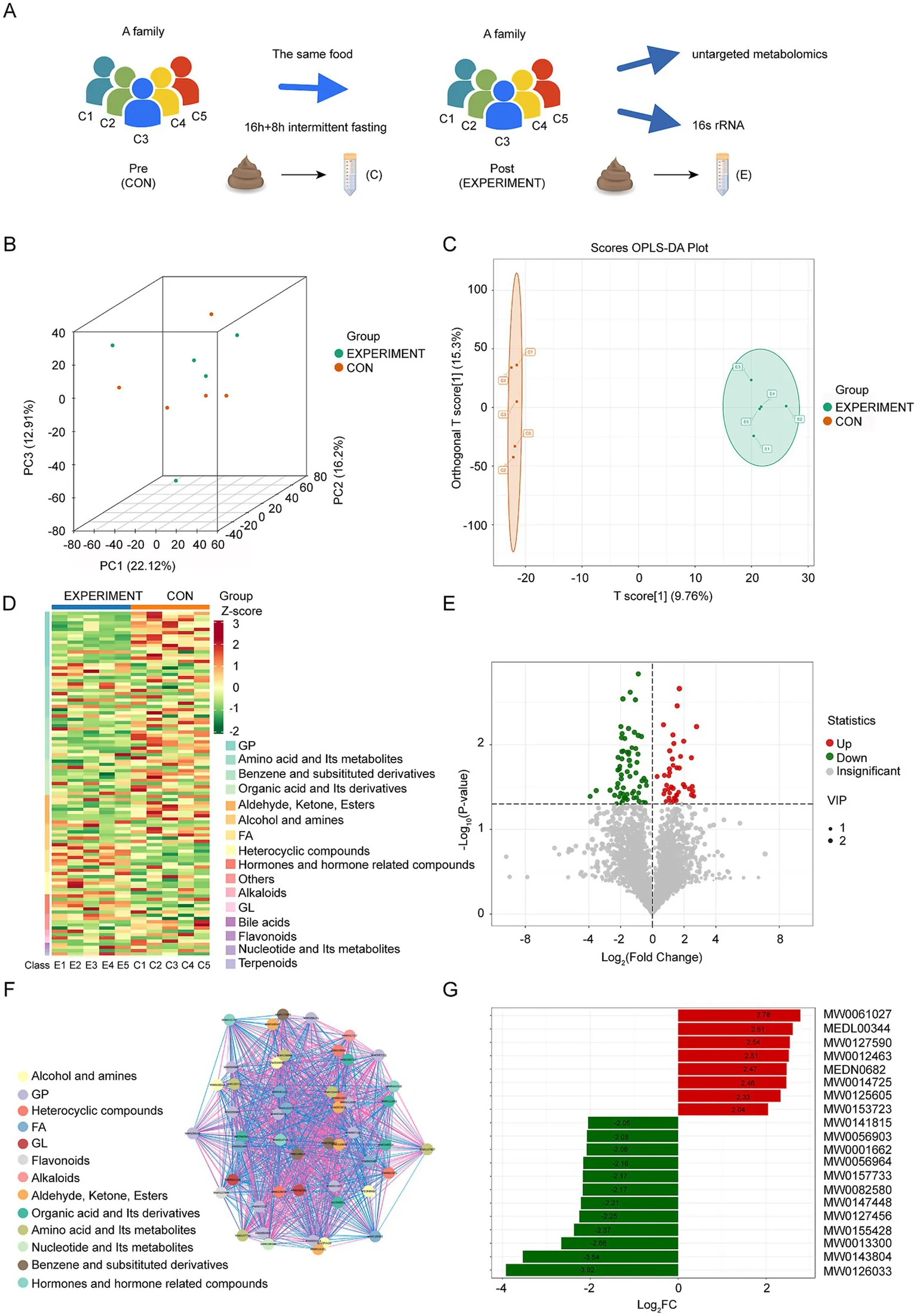
Intermittent fasting alters fecal metabolomic profiles in human subjects. **(A)** Schematic diagram of longitudinal fecal sampling for non-targeted metabolomics and 16S rRNA sequencing before and after intermittent fasting. **(B)** 3D score plot of principal component analysis (PCA; C: *n* = 5, E: *n* = 5). **(C)** Orthogonal partial least squares-discriminant analysis (OPLS-DA; C: *n* = 5, E: *n* = 5). **(D)** Clustering heatmap of differential metabolites (C: *n* = 5, E: *n* = 5). **(E)** Volcano plot of differential metabolites. **(F)** Correlation network of differential metabolites. **(G)** Bar plot of differential metabolites.

**Table 1:** Screening results of differential metabolites between baseline and post-IF.

Index	Compounds	Type
MEDN0682	Octadecanamide	Up
MW0015782	Androstanedione	Up
MW0154482	N-hydroxy-L-valine	Up
MW0052460	Docosatrienoic	Up
MW0059173	Peimine	Up
MEDP2105	3,6,9,12,15,18,21-Heptaoxatricosane-21,23-diol	Down
MADP0489	Heptethylene glycol	Down
MW0011902	1,2-Dioleoyl-sn-Glycero-3-Phosphocholine	Down
MW0056964	1-Stearoyl-2-arachidonoyl-sn-glycero-3-phosphocholine	Down
MW0147448	Cholesterol beta-epo	Down

Subsequently, we performed correlation analysis on the screened differential metabolites. The results demonstrate that IF-induced metabolic alterations constitute a complex regulatory network, characterized by extensive interconnections and mutual influences among various metabolites. For instance, changes in organic acids and their derivatives, as well as amino acids and their metabolites, may collectively participate in the regulation of energy metabolism during IF (Figure 2F). Notably, we observed numerous significant changes in energy metabolism-related metabolites following the IF intervention (Supplementary Figure 1A). The metabolomic data showed a relative increase in lipid-related metabolites compared to carbohydrate-related metabolites (Figure 2G). These results collectively indicate that IF intervention effectively modulates host energy metabolism. It should be noted that these findings are derived from fecal metabolomics, which primarily reflects gut luminal metabolism and microbial activity rather than direct systemic host metabolic parameters. Direct measurement of circulating metabolic markers (e.g., glucose, free fatty acids, triglycerides, insulin) was not performed in human subjects and represents an important direction for future validation.

### 3.3 Intermittent fasting induces multifaceted metabolic and functional remodeling

To elucidate the potential functional impacts of differential metabolites identified before and after intermittent fasting, we performed KEGG pathway enrichment analysis on the screened metabolites. Our analysis revealed that these differential metabolites significantly influenced organic systems (e.g., endocannabinoid signaling), metabolic pathways, and pathways related to human diseases and cellular processes (Figure 3A). Further cluster analysis demonstrated the multi-target molecular mechanisms through which IF intervention exerts its health benefits: by modulating the endocannabinoid system, promoting lipolysis and thermogenesis, and switching energy substrates to regulate core energy balance (32, 33); significantly affecting the metabolism of various lipids (phospholipids, unsaturated fatty acids, cholesterol); initiating autophagy to promote cellular renewal and activate cellular cleanup; and potentially preventing diseases by impacting pathways associated with atherosclerosis and insulin resistance (Figure 3B; Supplementary Figures 2 A,B).

**Figure 3:**
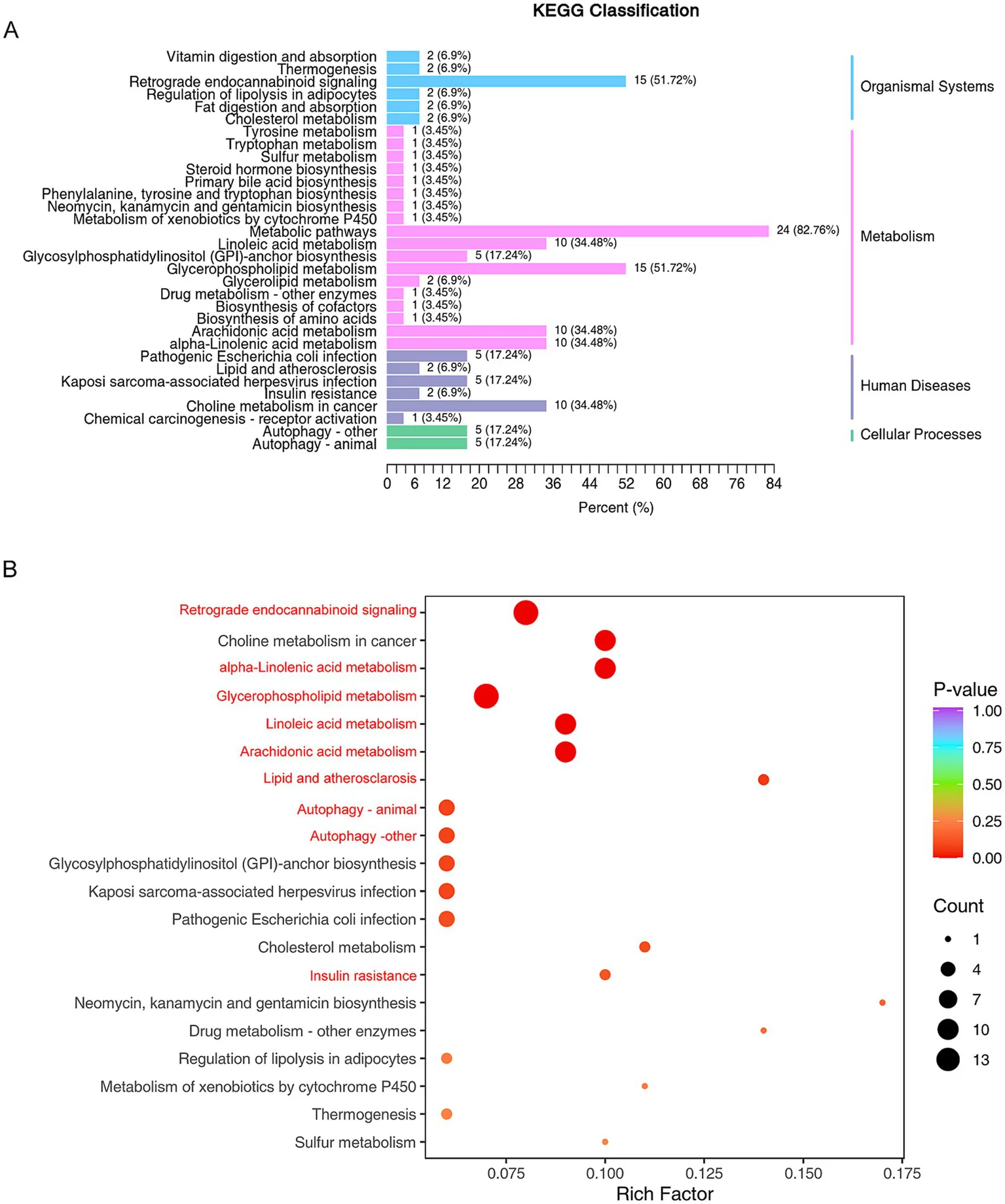
Intermittent fasting induces multifaceted metabolic and functional remodeling in human subjects. **(A)** Category plot of KEGG pathways for differential metabolites. **(B)** Enrichment plot of KEGG pathways for differential metabolites.

We further conducted HMDB functional annotation and enrichment on the differential metabolites. The results indicated significant alterations in intestinal immunity and inflammatory responses, evidenced by significant enrichment of terms such as the diphenhydramine H1-antihistamine action. Concurrently, metabolic pathways including “Alpha Linolenic Acid and Linoleic Acid Metabolism” and “Androstenedione Metabolism” also showed high enrichment levels and significance. Alpha-linolenic acid and linoleic acid are essential fatty acids known to effectively ameliorate intestinal inflammation and cardiovascular diseases (Figure 4A) (34). These findings are consistent with previous reports on IF interventions.

**Figure 4:**
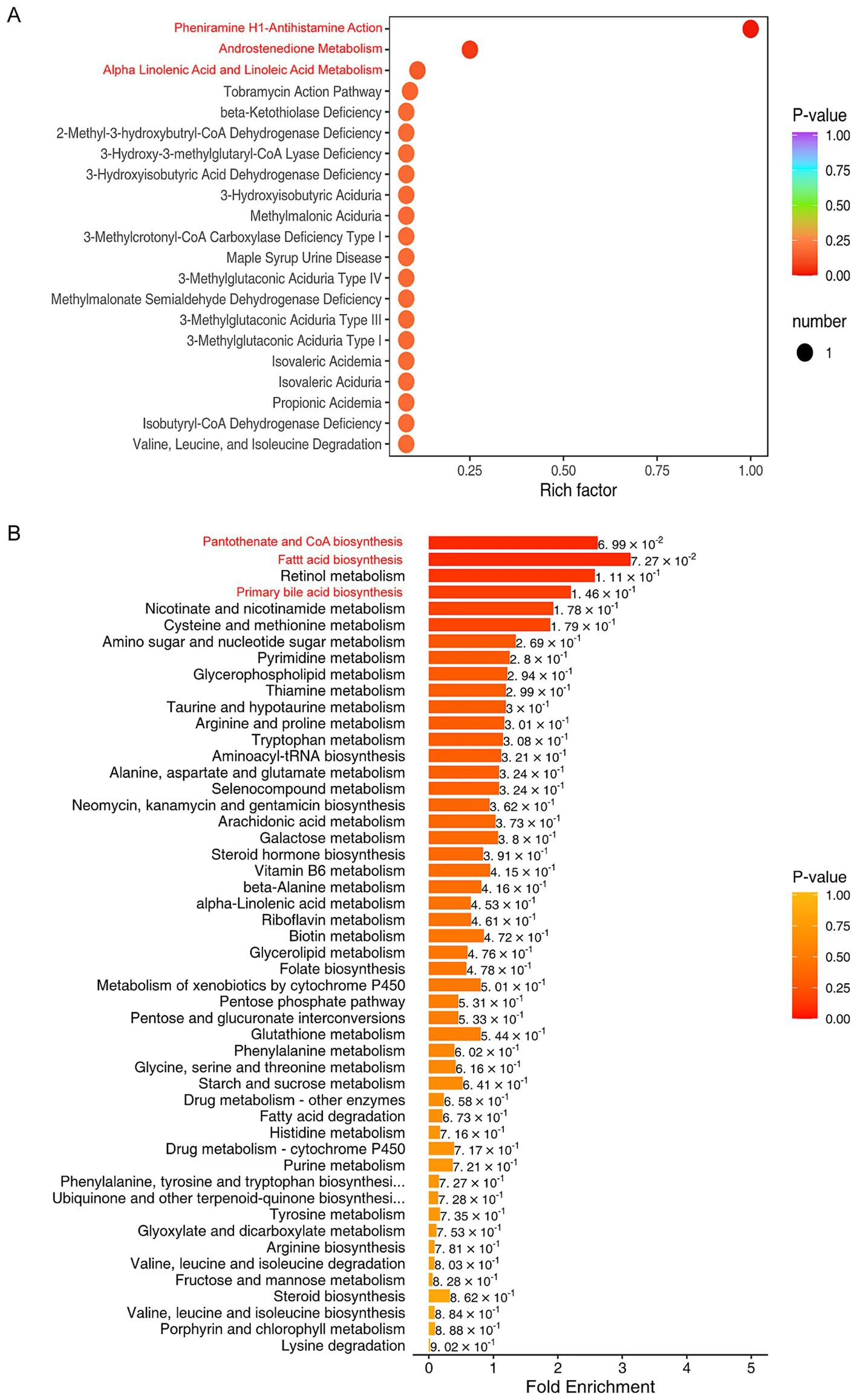
Intermittent Fasting Induces Multifaceted Functional Remodeling in Human Subjects. **(A)** Functional Annotation and Enrichment of Differential Metabolites in HMDB. **(B)** Metabolite Set Enrichment Analysis (MSEA) Plot.

Additional MSEA enrichment analysis identified significant enrichment in “Pantothenate and CoA Biosynthesis.” Pantothenate is a precursor of coenzyme A, which plays vital roles in various metabolic reactions, such as fatty acid metabolism and ATP synthesis (35, 36). The enrichment of this pathway suggests alterations in related metabolic activities within the gut and their association with the metabolism and utilization of nutrients. Interestingly, “Primary Bile Acid Biosynthesis” was also significantly enriched. Bile acids are crucial for fat digestion and absorption and also function in regulating the gut microbial community and host metabolism (37, 38). The enrichment of this biosynthesis pathway is linked to intestinal digestive function, lipid metabolism, and interactions with gut microbiota (Figure 4B). In summary, our findings indicate that the health benefits of IF are mediated by a complex reprogramming of host metabolic and inflammatory pathways, coupled with altered microbial metabolism.

### 3.4 IF-induced gut microbiota remodeling features reduced F/B ratio and community restructuring

To investigate alterations in the gut microbiota before and after intermittent fasting under standardized dietary conditions, we performed 16S rRNA gene sequencing on fecal samples collected pre- and post-intervention. Analysis of beta-diversity revealed relatively short within-group distances and greater between-group distances (Figures 5A,B), indicating differences in community structure between the two sampling time points. Further analysis showed partial separation of pre- and post-IF samples in the PCA plot, providing preliminary evidence of microbiome remodeling over the intervention period (Figure 5C). Concurrently, 657 amplicon sequence variants (ASVs) were shared between the two groups, indicating a substantial stable core of the microbial community (Figure 5D). Because this pilot cohort did not include a time-matched non-fasting control group, these longitudinal differences are interpreted as IF-associated rather than as effects attributable exclusively to fasting rhythm.

**Figure 5:**
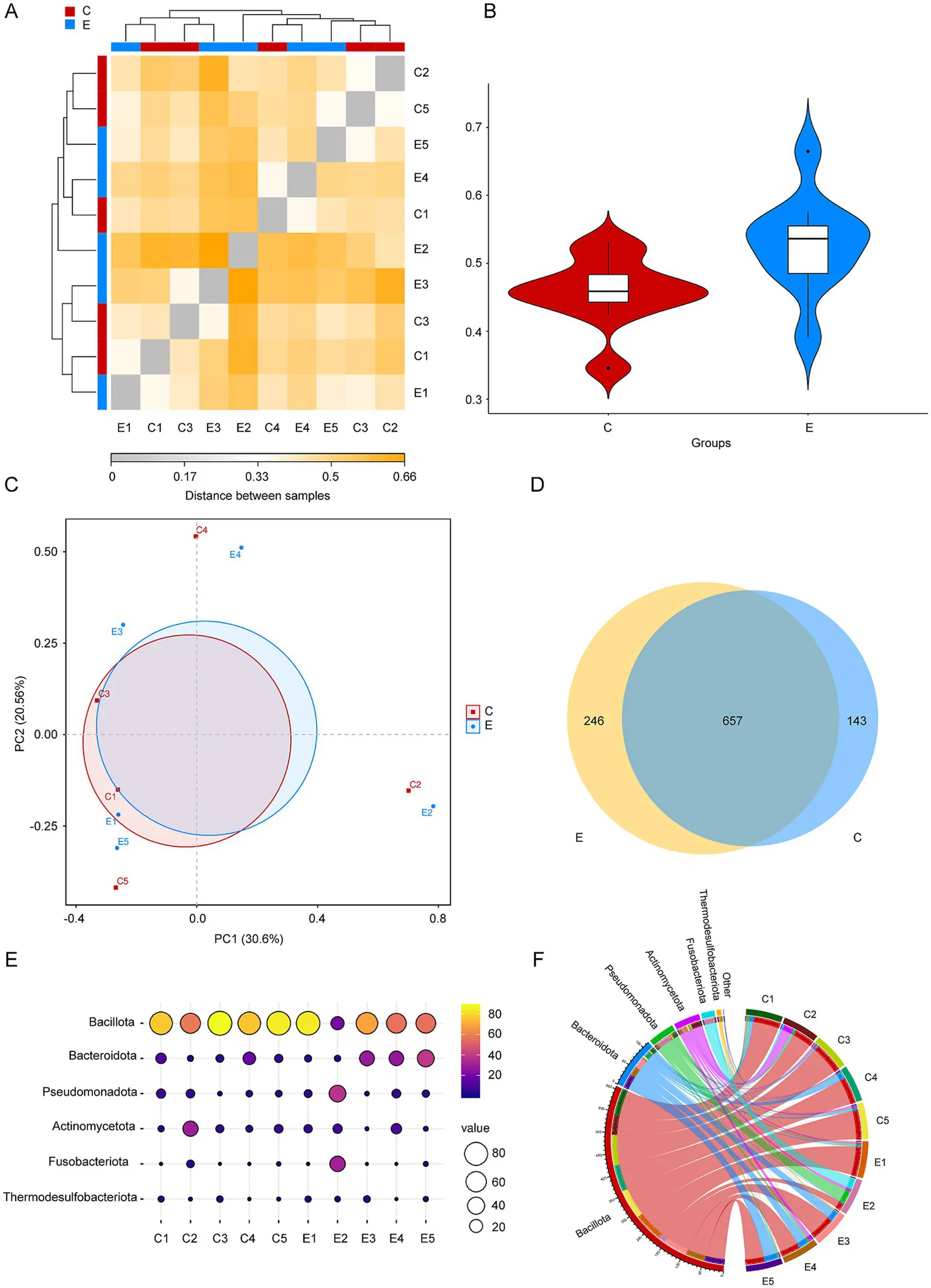
IF-induced gut microbiota remodeling features reduced F/B ratio and community composition changes. **(A)** Inter-sample distance heatmap between baseline and post-IF. (C: *n* = 5, E: *n* = 5). **(B)** Box plot of inter-sample distances between baseline and post-IF. (C: *n* = 5, E: *n* = 5). **(C)** Principal component analysis (PCA) score plot of baseline and post-IF. (C: *n* = 5, E: *n* = 5). **(D)** Venn diagram of species distribution between baseline and post-IF. (C: *n* = 5, E: *n=*5). **(E)** Bubble Plot of Species Abundance. **(F)** Co-linearity Network Diagram.

At the phylum level, Bacillota (Firmicutes) decreased and Bacteroidota increased after the intervention. Previous studies suggest that these phyla participate in distinct aspects of dietary substrate utilization and energy metabolism (39, 40). The observed pattern was accompanied by a lower Firmicutes-to-Bacteroidota (F/B) ratio and an increase in Actinobacteria (Figure 5E). Co-linearity analysis further illustrated changes in the proportional abundance of dominant taxa across samples (Figure 5F). Together, these descriptive findings indicate longitudinal remodeling of gut microbial community composition during the 4-week IF intervention; given the pilot design and absence of a time-matched non-fasting control group, they should be interpreted as hypothesis-generating associations rather than definitive fasting-specific effects.

### 3.5 Taxonomic and predicted functional changes in the gut microbiome after intermittent fasting

To evaluate genus-level microbiome changes while accounting for the longitudinal study design, we reanalyzed raw count data using a paired DESeq2 model including participant identity as a blocking factor (design = ~ subject + condition). No genus remained statistically significant after Benjamini-Hochberg correction (adjusted *p* < 0.05; complete results in Supplementary Table S1). Lacticaseibacillus retained a directional decrease after IF (log2FC = −2.55, SE = 2.09, Wald statistic = −1.22, *p* = 0.223, adjusted *p* = 0.992; Table 2; Figure 6A). Examination of the raw genus-level counts showed detectable Lacticaseibacillus reads in two of five participants at baseline and none after IF (Supplementary Figure 4). In the original unpaired DESeq2 analysis, Lacticaseibacillus represented the strongest differential signal (log2FC = −24.25, *p* = 1.12 × 10^−15^, adjusted *p* = 2.35 × 10^−13^; Supplementary Table S2), with a direction of change consistent with the primary paired analysis. However, because this model did not account for repeated measurements within participants, these results are retained for exploratory sensitivity comparison only and are not used for primary statistical inference.

**Table 2:** Selected results from the primary paired genus-level DESeq2 analysis (post-IF versus pre-IF; design = ~ subject + condition).

Genus	baseMean	log2FC	SE	*p* value	Adjusted p	Interpretation
Lacticaseibacillus	76.67	−2.55	2.09	0.2227	0.9923	Candidate; not FDR-significant
unclassified_Cyanobacteriia	33.80	−3.79	1.14	0.0009264	0.2464	Not FDR-significant
norank_Oscillospirales	5430.65	−2.63	1.14	0.02151	0.9923	Not FDR-significant
Clostridium	551.47	−2.51	1.09	0.02179	0.9923	Not FDR-significant
norank_Ruminococcaceae	1145.84	2.27	1.12	0.04347	0.9923	Not FDR-significant
Haemophilus	410.47	−2.52	1.29	0.05169	0.9923	Not FDR-significant

No genus reached adjusted *p* < 0.05. Complete paired DESeq2 results are provided in Supplementary Table S1. The complete original unpaired analysis is retained as an exploratory sensitivity analysis in Supplementary Table S2.

**Figure 6:**
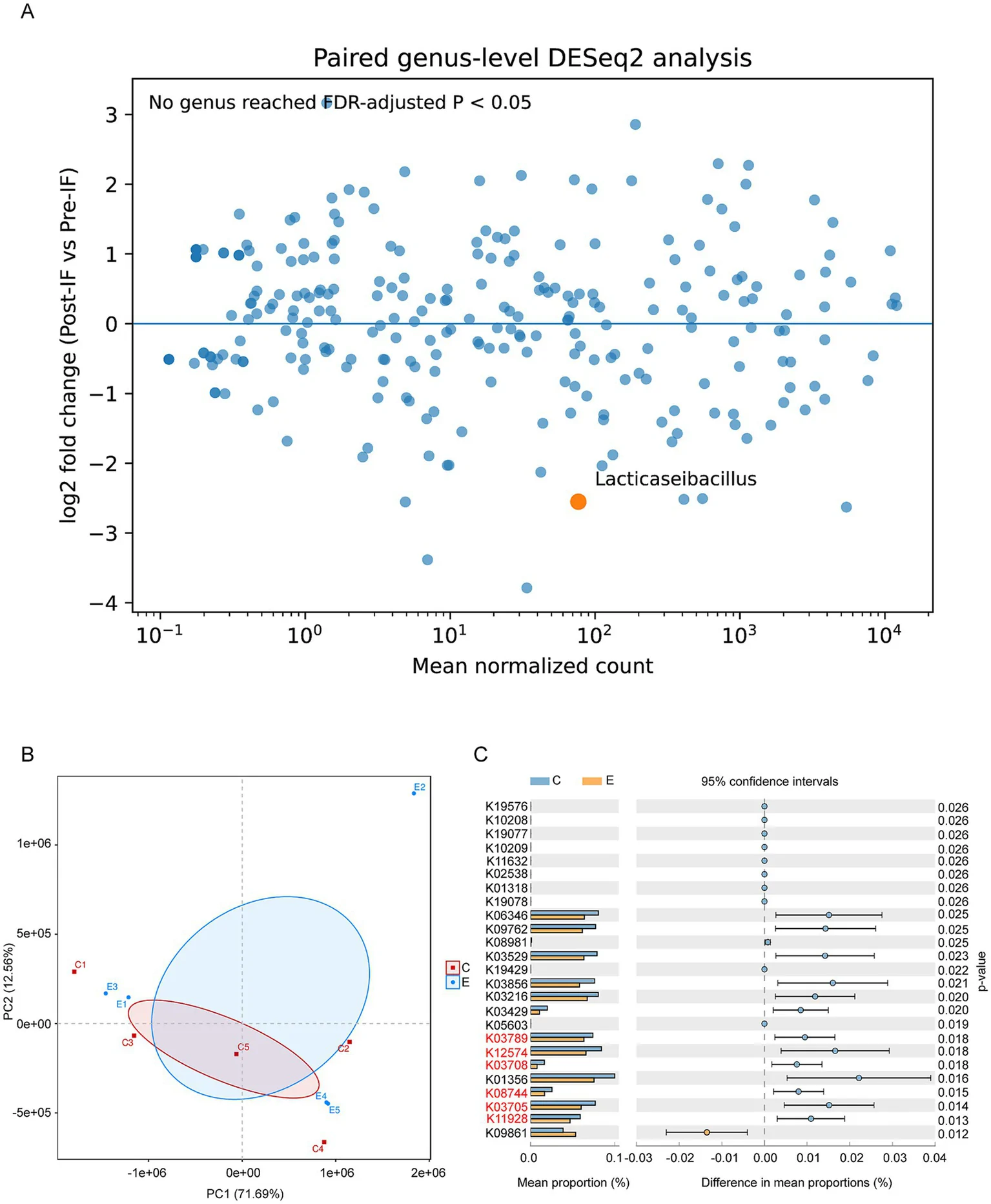
Taxonomic and predicted functional changes in the gut microbiome after intermittent fasting in human subjects. **(A)** MA plot from the primary paired DESeq2 analysis (design = ~ subject + condition). No genus reached FDR < 0.05; *Lacticaseibacillus* is highlighted as an exploratory candidate (log2FC = −2.55, *p* = 0.223, adjusted *p* = 0.992). **(B)** Functional principal component analysis (PCA) plot. (C): *n* = 5, (E) *n* = 5. **(C)** Predicted functional differential analysis of C versus E. Functional predictions are inferred from 16S taxonomic profiles.

Lacticaseibacillus is a lactic-acid-producing, carbohydrate-fermenting genus (41, 42), providing a biological rationale for examining whether Lacticaseibacillus-associated functions might relate to the broader metabolic patterns observed after IF. Functional prediction analysis revealed separation of pre- and post-IF profiles and changes in predicted pathways related to ATP and carbohydrate metabolism (Figures 6B,C; Supplementary Figure 3). These predictions are based on 16S-derived taxonomic profiles and therefore represent inferred functional potential rather than direct measurements of gene expression, enzymatic activity, fermentation flux, intestinal energy absorption, or whole-body energy expenditure. Together with the fecal metabolomic changes, these observations generated the hypothesis that altered microbial energy metabolism may contribute to the host response to IF. We therefore selected *L. rhamnosus* HN001 as a representative Lacticaseibacillus strain for functional testing in mice; this choice was intended as a mechanistic probe and should not be interpreted as evidence that HN001, or *L. rhamnosus* specifically, was the taxon altered in the human cohort.

### 3.6 Lacticaseibacillus rhamnosus HN001 modulates intermittent fasting-induced metabolic adaptation and attenuates weight loss

The directional human Lacticaseibacillus signal, together with predicted functional and metabolomic changes related to energy metabolism, motivated an experimental test of functional sufficiency in mice. We administered Lacticaseibacillus rhamnosus HN001 during IF as a representative Lacticaseibacillus strain with carbohydrate-fermenting and lactate-producing capacity. This experiment was designed to determine whether supplementation could modify the metabolic response to fasting, not to establish that depletion of this specific strain or species occurred in humans.

Previous studies have reported that mice have a higher metabolic rate than humans (43). To simulate 1 month of intermittent fasting in humans, mice were assigned to four groups (n = 6 per group): normally fed controls (Ctrl), normally fed mice receiving daily HN001 supplementation (Ctrl+HN001), mice undergoing 1 week of intermittent fasting (IF), and mice undergoing intermittent fasting with daily HN001 supplementation (IF+HN001; Figure 7A). The Ctrl+HN001 group was included to determine whether HN001 exerted metabolic effects independently of fasting. To assess whether the weight changes observed in IF-treated mice were accompanied by alterations in caloric intake, we measured daily chow consumption at the cage level throughout the 7-day intervention period. Daily food intake per cage was comparable across the four groups (Figure 7E). IF mice exhibited significant body-weight loss and elevated plasma βOHB compared with Ctrl mice, whereas HN001 supplementation during IF reversed both responses (Figures 7B,C). In contrast, HN001 supplementation under normal feeding conditions did not significantly alter body weight, plasma βOHB, plasma PYY, or food intake relative to Ctrl mice (all *p* > 0.05; Figures 7B–E). Thus, under the conditions tested, HN001 did not produce comparable metabolic changes in normally fed mice but modified the metabolic response to fasting. Plasma PYY did not differ significantly in the reported group comparisons (Figure 7D).

**Figure 7:**
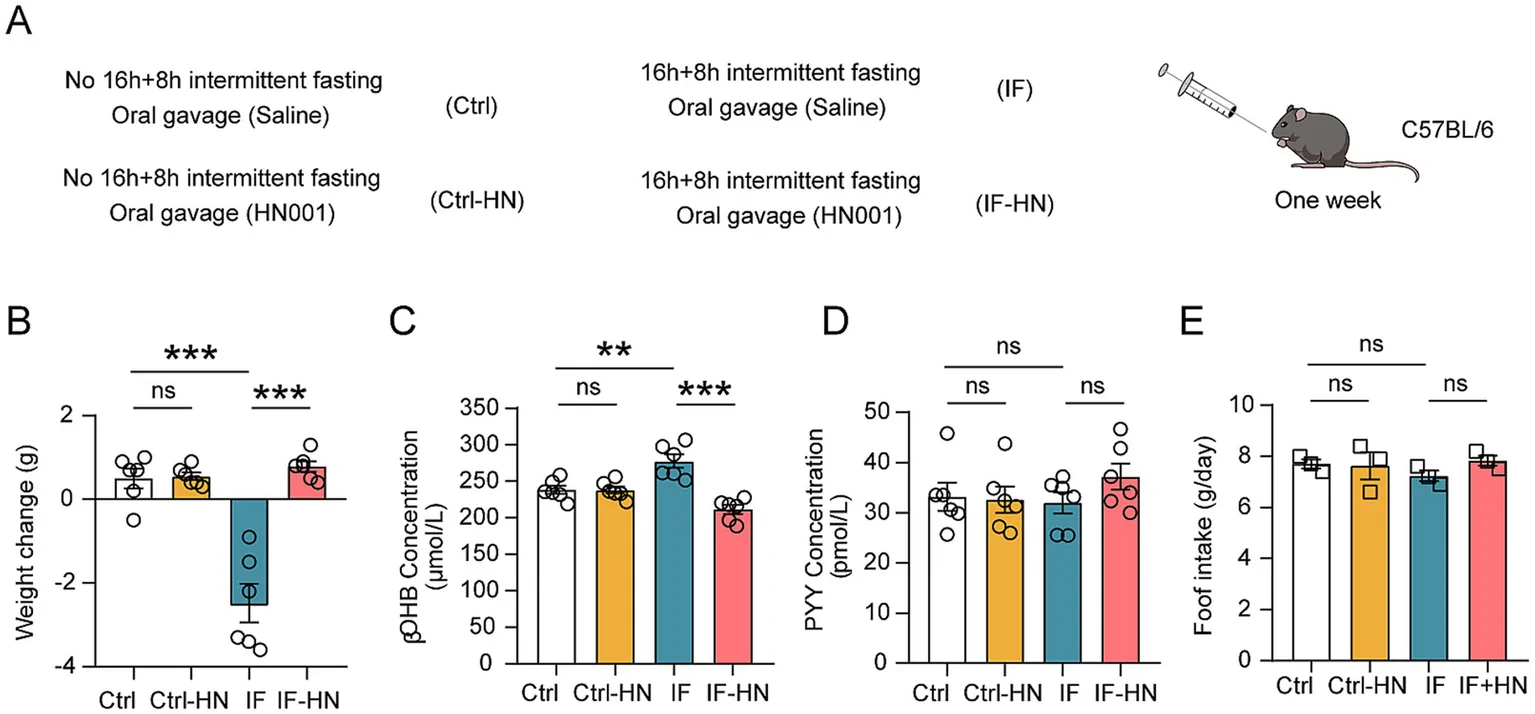
*Lacticaseibacillus rhamnosus* HN001 modulates the metabolic response to intermittent fasting. **(A)** Schematic diagram of the four-group mouse experiment: Ctrl, Ctrl+HN001, IF, and IF+HN001. **(B)** Body weight change after the 1-week intervention (*n* = 6 mice per group; Ctrl vs. Ctrl+HN001: unpaired *t* test, *p* = 0.8438, *t* = 0.2022; Ctrl vs. IF: unpaired *t* test, ***p = 0.0002, *t* = 5.818; IF vs. IF+HN001: Welch’s *t* test, ***p = 0.0005, *t* = 6.853). **(C)** Plasma βOHB concentration (*n* = 6 mice per group; Ctrl vs. Ctrl+HN001: unpaired *t* test, *p* = 0.9752, *t* = 0.03191; Ctrl vs. IF: unpaired *t* test, **p = 0.0046, *t* = 3.629; IF vs. IF+HN001: unpaired *t* test, ***p = 0.0001, *t* = 5.985). **(D)** Plasma PYY concentration (*n* = 6 mice per group; Ctrl vs. Ctrl+HN001: unpaired *t* test, *p* = 0.8810, *t* = 0.1536; Ctrl vs. IF: unpaired *t* test, *p* = 0.7398, *t* = 0.3414; IF vs. IF+HN001: unpaired *t* test, *p* = 0.1541, *t* = 1.542). **(E)** Daily food intake per cage (g/cage/day) in Ctrl, Ctrl+HN001, IF, and IF+HN001 groups. Food intake was measured at the cage level (*n* = 3 cages per group, 2 mice per cage), and each data point represents the 7-day average for a single cage; the cage was treated as the experimental unit. No reported comparison was significant (Ctrl vs. Ctrl+HN001: unpaired *t* test, *p* = 0.9116, *t* = 0.1183; Ctrl vs. IF: unpaired *t* test, *p* = 0.1550, *t* = 1.750; IF vs. IF+HN001: unpaired *t* test, *p* = 0.1045, *t* = 2.092).

Importantly, these findings reflect the acute phase of IF (1 week in mice), a period of rapid metabolic adaptation; the persistence of this effect during chronic IF warrants further investigation. We did not profile the murine gut microbiota to determine whether IF altered endogenous Lacticaseibacillus abundance because the mouse experiment was designed specifically as a functional supplementation test. HN001 supplementation significantly rescued IF-induced weight loss (Figure 7B) and reduced the IF-induced increase in plasma betaOHB to a level comparable to Ctrl mice (Figure 7C). These findings demonstrate that HN001 supplementation is sufficient to modify the acute metabolic response to IF under the conditions tested, while not establishing that endogenous *L. rhamnosus* depletion is necessary for the phenotype. Plasma PYY remained unchanged across groups (Figure 7D).

## 4 Discussion

Our findings are consistent with the hypothesis that gut microbiota remodeling and functional reprogramming contribute to the metabolic response to intermittent fasting (IF). In the pilot human cohort, paired genus-level analysis did not identify any taxon that remained significant after FDR correction; Lacticaseibacillus instead emerged as a directional, hypothesis-generating candidate. The accompanying predicted functional and fecal metabolomic changes were consistent with altered microbial energy metabolism. The controlled four-group mouse experiment provided a complementary functional test of *L. rhamnosus* HN001. HN001 supplementation under normal feeding conditions did not significantly alter body weight, plasma βOHB, PYY, or food intake relative to Ctrl mice, whereas supplementation during IF reversed the IF-associated changes in body weight and βOHB. This pattern supports the interpretation that HN001 modifies the metabolic response to fasting rather than producing a comparable metabolic phenotype under normal feeding conditions. These two evidence levels should be distinguished: the human data are exploratory associations, whereas the mouse data provide experimental evidence that HN001 supplementation can alter the metabolic response to IF under the conditions tested.

The family-based shared-meal design provided unusually tight control of dietary composition, reducing a major source of variation in microbiome studies of intermittent fasting (44). This feature strengthens within-cohort interpretation of longitudinal changes during the intervention. At the same time, the design should not be viewed as fully isolating the causal effect of feeding rhythm: the human cohort consisted of only five members of a single family and lacked a time-matched non-fasting control group. Accordingly, temporal variation, adaptation to the standardized diet, and other unmeasured behavioral or environmental factors cannot be completely separated from the IF intervention. We therefore interpret the human component as a tightly controlled pilot study that generates mechanistic hypotheses for subsequent testing rather than as a definitive causal trial.

The observed reduction in the Firmicutes/Bacteroidota ratio reflects a shift in microbial community composition that has been associated with leanness in some studies (39, 40). However, the F/B ratio is context-dependent and should not be interpreted as a universal biomarker of metabolic health. More importantly, predicted microbial functions related to ATP synthesis and carbohydrate metabolism changed across the intervention. Because these functions were inferred from 16S-derived taxonomic profiles, they indicate predicted functional potential rather than direct measurements of microbial gene expression, fermentation flux, intestinal energy absorption, or whole-body energy expenditure. Together with the fecal metabolomic data, these patterns support a hypothesis of reduced microbial energy extraction during IF. Within this framework, the directional Lacticaseibacillus signal is considered a candidate component of the broader ecological shift, not a statistically established keystone event in the human cohort.

A central observation of this study is the directional Lacticaseibacillus signal in the pilot human cohort and its convergence with predicted functional and metabolomic changes related to energy metabolism. Importantly, the paired DESeq2 analysis did not support statistically significant genus-level depletion after FDR correction, and V3-V4 sequencing cannot establish that the human signal represents *L. rhamnosus* specifically. The mouse experiment therefore serves as a functional sufficiency test rather than direct validation of a species-specific human depletion event. Importantly, HN001 supplementation under normal feeding conditions did not significantly alter the measured metabolic endpoints relative to Ctrl mice, whereas supplementation during IF reversed IF-associated weight loss and ketosis. This pattern supports a fasting-context-dependent effect of HN001 on metabolic adaptation. These findings support the biological plausibility that Lacticaseibacillus-associated functions can modulate the metabolic response to fasting, but they do not establish that endogenous *L. rhamnosus* depletion is necessary for the phenotype.

It is noteworthy that under strictly controlled dietary conditions, the present study observed no change in plasma PYY in mice following intermittent fasting, differing from reports in some studies without comparable dietary control (45–47). In the human pilot cohort, body weight decreased by 2.1 kg over 4 weeks without a significant reduction in reported caloric intake, while microbiome functional predictions and fecal metabolomics were consistent with altered substrate utilization. This dissociation is compatible with the hypothesis that changes in microbial energy extraction may contribute to the early metabolic response to IF. However, because the human study lacked direct measurements of energy expenditure, thermogenesis, physical activity, fecal energy loss, and a time-matched non-fasting control group, this mechanism cannot be established from the human data alone. The controlled mouse supplementation experiment provides an independent functional test showing that HN001 can modify IF-induced weight loss and ketosis, while the persistence and relevance of this mechanism during longer-term IF remain to be determined.

Beyond its role in energy metabolism, *L. rhamnosus* has been extensively investigated for effects on host physiology, including gut-brain signaling (48–51). In the present study, the human data identify an exploratory genus-level Lacticaseibacillus signal rather than significant or species-specific depletion of *L. rhamnosus*. In mice, HN001 supplementation reversed IF-induced weight loss under the conditions tested. These findings raise the possibility that manipulating Lacticaseibacillus-associated functions could influence host energy handling, but they do not support clinical recommendations. In particular, any potential application to pathological weight loss, obesity, or other clinical settings remains speculative and would require dedicated studies in appropriately powered human cohorts.

A critical translational question is how the exploratory human observations relate to the controlled mouse intervention. The human and mouse experiments address different levels of evidence. In the pilot human cohort, paired genus-level analysis did not identify FDR-significant taxa, although Lacticaseibacillus showed a directional decrease that was consistent with the original exploratory unpaired analysis. The accompanying predicted functional and fecal metabolomic changes generated the hypothesis that Lacticaseibacillus-associated functions may participate in IF-related metabolic remodeling. The four-group mouse experiment then tested functional sufficiency. HN001 supplementation produced no significant changes in the measured metabolic endpoints under normal feeding conditions, but reversed IF-associated weight loss and ketosis when administered during fasting. This context-dependent pattern provides experimental evidence that HN001 can modulate the metabolic response to IF, but it does not establish that endogenous *L. rhamnosus* depletion occurs in humans or mice, that such depletion is necessary for the phenotype, or that the same mechanism operates identically across species. Direct species-resolved microbiome measurements, depletion/reconstitution experiments, and matched systemic metabolic phenotyping will be required to establish those links.

Several limitations should be considered. First, the human component was a pilot, hypothesis-generating cohort of only five participants from a single family (3 males, 2 females). This highly homogeneous shared-meal design reduced variation in dietary composition and environmental exposure, but the small sample size limits statistical power, precludes meaningful sex-stratified analyses, and restricts generalizability beyond this family. In addition, there was no time-matched non-fasting control group. Consequently, temporal variation, adaptation to the standardized diet, behavioral changes, environmental factors, or regression to the mean cannot be excluded as alternative explanations for some longitudinal changes. The human findings should therefore be interpreted as exploratory associations that require replication in larger, genetically unrelated cohorts with appropriate control groups. Second, predicted microbial functions were inferred from 16S profiles rather than measured directly by metatranscriptomics or metaproteomics. Third, body composition was not measured in humans or mice, so the tissue source of weight change cannot be determined. Fourth, human metabolic phenotyping relied on fecal metabolomics without direct longitudinal blood measurements of glucose, insulin, free fatty acids, or betaOHB. Fifth, although the four-group mouse design showed that HN001 supplementation modified the fasting-associated phenotype without producing comparable changes under normal feeding conditions, the experiment did not determine whether IF depletes endogenous *L. rhamnosus* in mice, whether such depletion is necessary for the phenotype, or whether the observed effect reflects direct microbial, metabolite-mediated, or host-mediated mechanisms. Sixth, V3-V4 16S sequencing cannot definitively resolve closely related Lacticaseibacillus species, and no species-specific qPCR, shotgun metagenomics, culture-based validation, or absolute bacterial quantification was performed. In the primary paired DESeq2 analysis, Lacticaseibacillus did not remain significant after FDR correction, and raw genus-level reads were detectable in two of five baseline samples and none of the post-IF samples; this taxon-level observation is therefore hypothesis-generating. Seventh, physical activity, sleep, stress, and other behavioral variables were not comprehensively assessed. Finally, 16S sequencing provides compositional relative-abundance data, so changes in relative abundance do not necessarily represent changes in absolute bacterial cell numbers.

The deliberate trade-off in the human study was therefore tighter dietary and household control at the expense of sample size, independence, and external validity. The study should be viewed as a proof-of-concept platform for identifying candidate microbiome-metabolic relationships under standardized feeding conditions. Whether the observed longitudinal patterns are reproducible in unrelated individuals, persist under free-living conditions, and are specifically attributable to IF rather than time or other co-occurring factors remains to be established in larger controlled studies.

In summary, the pilot human study identified coordinated longitudinal microbiome, predicted functional, and fecal metabolomic changes during a standardized 4-week IF intervention. The paired genus-level analysis did not identify taxa that remained significant after FDR correction, although Lacticaseibacillus showed a directional decrease that motivated subsequent functional testing. The mouse experiment provides a distinct and stronger level of experimental evidence: HN001 supplementation did not significantly alter the measured metabolic endpoints in normally fed mice but reversed IF-associated weight loss and ketosis when administered during fasting. Together, these findings support a context-dependent functional effect of HN001 on the acute metabolic response to IF while preserving the distinction between exploratory human associations and experimental functional evidence in mice.

## 5 Conclusion

This pilot, hypothesis-generating study suggests that intermittent fasting under standardized dietary conditions is associated with coordinated microbiome, predicted functional, and fecal metabolomic remodeling. In the human cohort, no genus remained significant after FDR correction in the primary paired DESeq2 analysis, although Lacticaseibacillus showed a directional decrease consistent with the original exploratory analysis. In mice, HN001 supplementation did not significantly alter the measured metabolic endpoints under normal feeding conditions but reversed IF-associated weight loss and ketosis during fasting, supporting a context-dependent functional effect on the acute IF phenotype. Larger independent human cohorts with time-matched non-fasting controls, species-resolved microbiome measurements, and targeted mechanistic studies are required to determine whether the candidate mechanism identified here generalizes to humans.

## Data Availability

The data of 16S rRNA gene sequencing and non-targeted metabolomics analysis in this study have been deposited in the Genome Sequence Archive under accession code CRA032255 (https://ngdc.cncb.ac.cn/gsa). All requests for raw data may be sent to the corresponding author.
