# Supplementary material for: Intermittent fasting-associated microbiome remodeling and functional modulation by Lacticaseibacillus rhamnosus HN001
Source: Front Nutr. 2026 Sep 24;13:1947132. doi: 10.3389/fnut.2026.1947132 (PMC13647467; doi:10.3389/fnut.2026.1947132)
Supplement: Supplementary file 1 [file Supplementary_file_1.DOCX]

**Supplementary material**

**Figure S1**. Numerous significant changes in metabolites following the IF intervention. (A) Violin Plot of Raw Values of C group and E group.

**Figure S2**. Intermittent Fasting Induces Multifaceted Metabolic Remodeling related to Figure 3. (A) Clustering Heatmap of Differential Metabolites across KEGG Pathways. (C: n=5, E: n=5). (B) KEGG Pathway Analysis with Differential Abundance (DA) Score Plot.

**Figure S3**. A comprehensive functional reprogramming of the gut microbiota in response to nutritional stress. (A) Functional Abundance Heatmap of C group and E group.

**Figure S4**. Individual-level genus-level relative abundance of Lacticaseibacillus before and after the 4-week IF intervention in five human participants. Each line represents one participant (n=5). Relative abundance is displayed on a log10 scale with a pseudocount of 1 × 10⁻⁵ to facilitate visualization of zero values. Lacticaseibacillus reads were detectable in 2/5 participants at baseline and 0/5 participants post-IF. Because V3-V4 16S sequencing does not permit definitive species-level resolution among closely related Lacticaseibacillus taxa, this analysis is interpreted at the genus level.
